# Alpha7 nicotinic acetylcholine receptor agonist promotes retinal ganglion cell function via modulating GABAergic presynaptic activity in a chronic glaucomatous model

**DOI:** 10.1038/s41598-017-02092-6

**Published:** 2017-05-11

**Authors:** Xujiao Zhou, Yun Cheng, Rong Zhang, Gang Li, Boqi Yang, Shenghai Zhang, Jihong Wu

**Affiliations:** 1 Eye & ENT Hospital, State Key Laboratory of Medical Neurobiology, Institutes of Brain Science and Collaborative Innovation Center for Brain Science, Shanghai Medical College, Fudan University, Shanghai, 200032 China; 2 Shanghai Key Laboratory of Visual Impairment and Restoration, Shanghai, 200032 China; 3 Key Laboratory of Myopia, Ministry of Health, Shanghai, 200032 China

## Abstract

Alpha-7 nicotinic acetylcholine receptor (α7-nAChR) agonists can prevent glutamate-induced excitotoxicity in cultured retinal ganglion cells (RGCs). However, the neuroprotective effects and the mechanism of action of PNU-282987, an α7-nAChR agonist, in a chronic *in vivo* rat glaucoma model are poorly understood. We found that elevated intraocular pressure (IOP) downregulated retinal α7-nAChR expression. Electroretinography revealed that the amplitude of the photopic negative response (PhNR) decreased in parallel with the loss of RGCs caused by elevated IOP. PNU-282987 enhanced RGC viability and function and decreased terminal deoxynucleotidyl transferase dUTP nick end labeling (TUNEL)-positive signals in RGCs. Patch-clamp recordings revealed differences in the baseline frequencies and decay times of the miniature GABAergic inhibitory postsynaptic currents (mIPSCs) of RGCs between control and glaucomatous retinal slices. The results of western blotting and immunostaining showed that glutamic acid decarboxylase 65/67 and GABA deficits persisted in glaucomatous retinas and that these deficits were reversed by PNU-282987. Patch-clamp recordings also showed that PNU-282987 significantly increased the frequency and amplitude of the GABAergic mIPSCs of RGCs. The protective effects of PNU-292987 were blocked by intravitreal administration of selective GABA_A_ receptor antagonists. The modulation of GABAergic synaptic transmission by PNU-282987 causes de-excitation of ganglion cell circuits and suppresses excitotoxic processes.

## Introduction

Glaucoma is an irreversible cause of blindness that is characterized by the progressive loss of retinal ganglion cells (RGCs) and eventually the visual field^1^. One remarkable characteristic of glaucoma is the progressive deterioration of RGCs^2^. Strategies that maximize the recovery of injured RGCs may prevent ongoing visual impairment in glaucoma.

There is ample evidence showing that drugs capable of potentiating cholinergic function, such as α7 nicotinic acetylcholine receptor (α7-nAChR) agonists, promote the survival of acutely isolated or cultured RGCs^3, 4^. In isolated/cultured porcine and salamander RGCs, the neuroprotective effects of nAChR agonists against glutamate-induced excitotoxicity are mediated by the Ca^2+^-phosphatidylinositol 3-Akt-Bcl2 and NF-κβ cell survival signaling pathway, and via the mitogen-activated protein kinase apoptosis pathway^5–11^. Previous studies have demonstrated that α7-nAChRs are expressed in retinal bipolar, amacrine, and ganglion cells in normal mice and rabbits^12–14^. However, it is unclear whether activation of α7-nAChRs exerts a neuroprotective role in a chronic *in vivo* rat glaucoma model produced by episcleral vein cautery.

The excitotoxic effects of overactivation of the glutamate receptor contribute to the pathology of glaucoma^2, 15–18^. This finding suggests that cell death/survival may depend on an appropriate balance between excitatory and inhibitory pathways^19^. Imbalances in these pathways may lead to severe retinal dysfunction and disease^20–24^. The inhibitory neurotransmitter GABA is another modulator of neural circuits. In the brain, GABAergic function is depressed following an ischemic insult, and increasing cerebral GABA concentrations decreases neuronal vulnerability to excitotoxic damage by increasing Cl^−^ flux across the postsynaptic neuron and by inhibiting NMDA-induced Ca^2+^ influx^25, 26^. In the retina, excitotoxicity leads to excessive synaptic excitation; however, few studies have investigated whether the GABAergic inhibitory effects on RGCs are abnormal in chronic glaucoma^22^. An earlier study revealed significant dysfunction of the retinal GABAergic system in rats with hyaluronic-acid-induced intraocular pressure (IOP) elevation^27^; another study reported significant loss of GABAergic amacrine cell immunoreactivity in DBA/2J mice with inherited glaucoma relative to normal C57BL/6J mice^28^. However, Quigley *et al*.^29^ found that the number of labeled GABAergic amacrine cells was not significantly affected in translimbal trabecular laser-induced glaucoma. Based on these findings, it is still controversial whether the GABAergic system is affected by glaucoma^30^.

Nicotinic receptors have been reported to play critical roles in the physiology and pathogenesis of the central nervous system^31–33^. In particular, α7-nAChR-knockout mice display decreased cortical levels of GABAergic markers^34–36^, suggesting that α7-nAChRs influence the synaptic GABAergic system in CNS dysfunction and disorders. Therefore, we conducted electrophysiological tests in rats to examine whether and how α7-nAChRs regulate GABAergic synaptic transmission in the inner retina.

In this study, we investigated whether a highly selective α7-nAChR agonist (PNU-282987) promotes RGC survival and functional recovery and examined its underlying mechanism of action in a rat model of chronic glaucoma. We showed that α7-nAChR mRNA and protein levels, the density of glutamic acid decarboxylase (GAD)65/67, and GABA levels were downregulated by ocular hypertension and that these effects of elevated IOP were prevented by PNU-282987. Using patch-clamp studies of rat retinal slices, we showed that PNU-282987 promotes GABA_A_ receptor-mediated miniature GABAergic inhibitory postsynaptic currents (mIPSCs) in RGCs.

## Results

### Ocular hypertension downregulates α7-nAChR expression in rat RGCs

**Figure 1:**
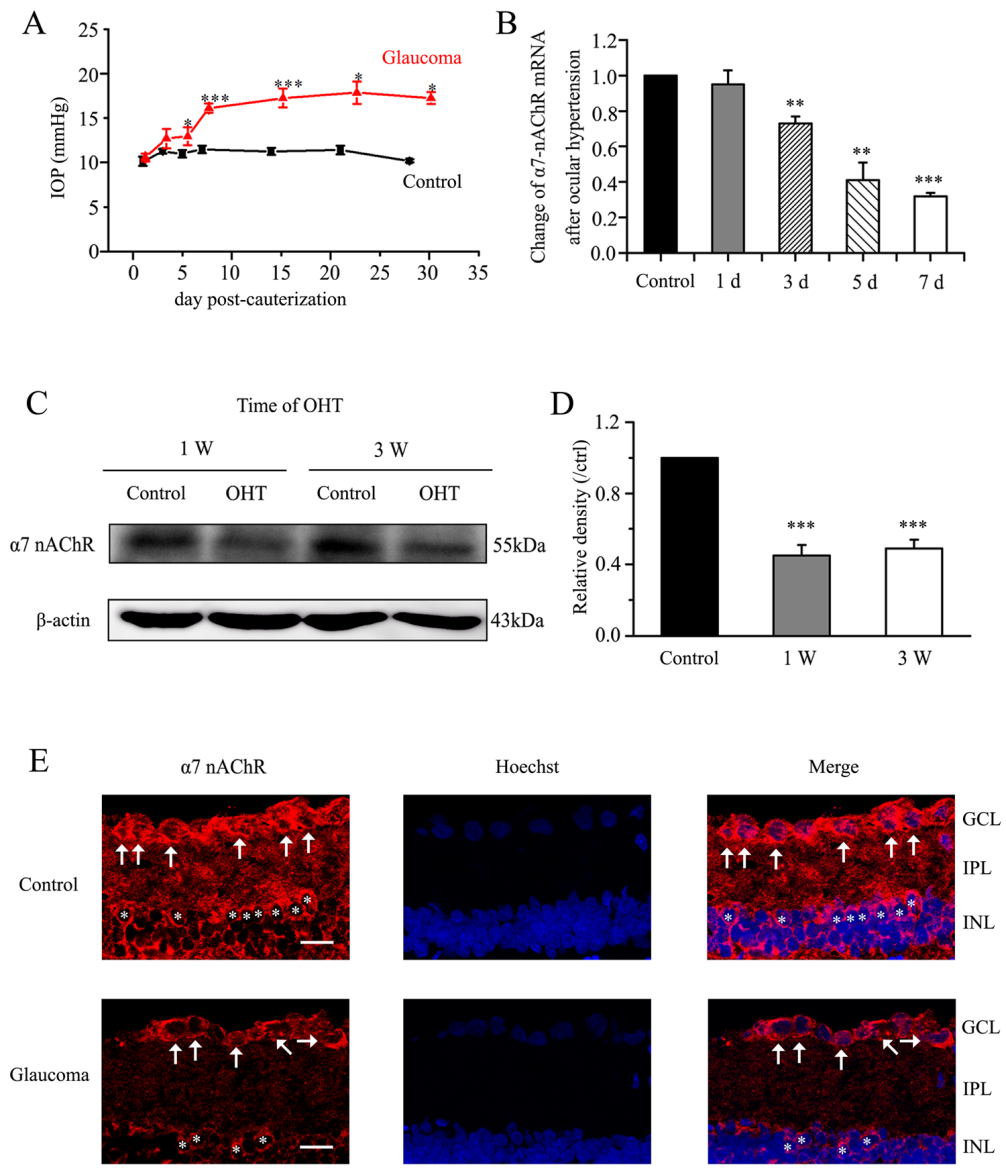
Chronic ocular hypertension downregulates α7-nAChR expression in adult rat retina. (**A**) Changes in IOP after inducing ocular hypertension. The IOP is significantly greater in eyes that underwent EVC than in control eyes (***p < 0.001). (**B**) Real-time PCR analysis of α7-nAChR mRNA expression in control and glaucomatous retinas at 1, 3, 5, and 7 days after EVC (1 day, *n* = 4; 3 days, *n* = 4; 5 days, *n* = 4; 7 days, *n* = 6); expression was normalized by the expression in the control retina. **p < 0.01, ***p < 0.001 vs. control retina. (**C**) Representative western blot of α7-nAChR expression in control and glaucomatous retinas. The expression of the 55 kDa protein decreases at 1 week after EVC and is sustained for up to 3 weeks. The full-length blots are presented in Supplementary Figure S1. (**D**) Densitometric analysis of α7-nAChR expression at 1 week (*n* = 6) and 3 weeks (*n* = 8) after EVC. Expression was normalized to the expression in the control retina. ***p < 0.001 vs. control retina. (**E**) Micrographs of 10 μm thick transverse sections of control and glaucomatous retinas. In contrast to the control retina (top panel), only a few ganglion cells are observed in the glaucomatous retina (bottom panel). Bar, 15 μm. The results in **A**, **B**, and **D** are expressed as the mean ± SE. α7-nAChR, α7 nicotinic acetylcholine receptor; d, day; IOP, intraocular pressure; OHT, ocular hypertension; w, week; GCL, ganglion cell layer; INL, inner nuclear layer; IPL, inner plexiform layer. The arrows indicate RGCs; the asterisks indicate amacrine cells.

Unilateral elevation of ocular hypertension was successfully induced in Wistar rats by episcleral vein cauterization (EVC)^37^ (Fig. 1A). The IOP (mean ± standard error (SE)) was significantly elevated at 3 weeks in EVC-treated eyes (17 ± 0.47 mmHg, *n* = 135) relative to control eyes (11.2 ± 0.22 mmHg, *n* = 135; *p* = 0.001), and this elevation was observed at all measurement times.

We first assessed whether α7-nAChR mRNA and protein levels were altered in glaucomatous rat retinas relative to control retinas. Quantitative RT-PCR (qRT-PCR) of retinal samples was performed at 1, 3, 5, and 7 days after EVC. The α7-nAChR mRNA level in EVC-treated eyes relative to control eyes (Fig. 1B) decreased to 95% ± 8% (mean ± SE) on day 1 (*n* = 4), to 73% ± 4% on day 3 (*n* = 4, *p* = 0.008), to 41% ± 10% on day 5 (*n* = 4, *p* = 0.010) and to 32% ± 2% on day 7 (*n* = 6, *p* = 0.001), confirming that ocular hypertension causes a gradual, time-dependent decrease in retinal α7-nAChR mRNA expression (Fig. 1B). Similarly, the results of western blotting showed marked decreases in α7-nAChR protein levels in glaucomatous retinas relative to control retinas, particularly at 1 week after EVC (Fig. 1C,D). Antibodies against α7-nAChR and β-actin recognized single bands at approximately 55 and 43 kDa. The mean α7-nAChR protein level decreased to 45% ± 6% (mean ± SE) of the control level at 1 week (*n* = 6, *p* = 0.001) and to 49% ± 5% of the control level at 3 weeks after EVC (*n* = 8, *p* = 0.001). As shown in Fig. 1E, α7-nAChR expression was localized to the ganglion cell layer (GCL), the inner plexiform layer (IPL) and the inner nuclear layer (INL) of the retinal section obtained from control retina. The fluorescent intensity of α7-nAChR expression was very low in these three layers of glaucomatous retinal section. The results of immunofluorescence revealed weak α7-nAChR staining in the glaucomatous retinas, consistent with the western blotting results. Collectively, these results provide evidence that chronic ocular hypertension downregulates retinal α7-nAChR mRNA and protein expression in adult rats.

### Activation of the α7-nAChR promotes RGC survival and ameliorates retinal dysfunction in experimental glaucoma

**Figure 2:**
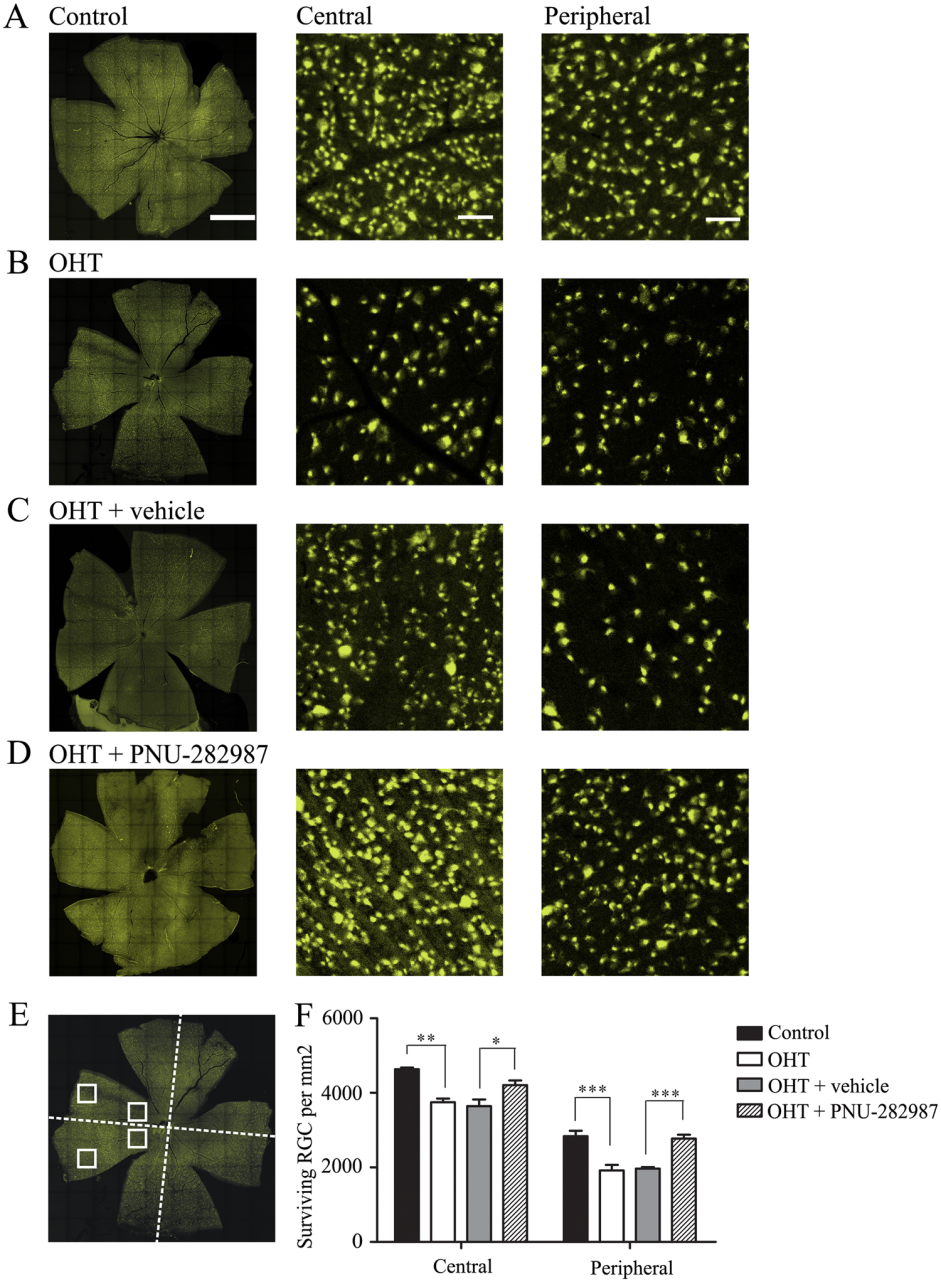
FluoroGold labeling of RGCs in normal and glaucomatous retinas. (**A–D**) FluoroGold labeling of surviving RGCs in flat-mounted retinas 3 weeks after inducing ocular hypertension. The left panels in **A–D** were photographed at low magnification (scale bar, 2 mm). Enlarged images (scale bar, 50 μm) from the same retinas are shown in the central (middle) and peripheral (right) panels. PNU-282987 increased RGC viability and preserved cellular integrity (**D**) relative to the vehicle (**C**). (**E,F**) Quantitative analysis of RGC survival in control eyes, ocular hypertension eyes with or without the vehicle and PNU-282987-treated ocular hypertension eyes (*n* = 10). *p < 0.05, **p < 0.01, and ***p < 0.001 (one-way analysis of variance). The results are expressed as the mean ± SE. OHT, ocular hypertension; RGC, retinal ganglion cell.

To examine whether upregulated α7-nAChR activity promotes RGC survival, we counted the number of FluoroGold-labeled RGCs in flat-mounted retinas. Consistent with previous studies, the number of retinal RGCs was significantly reduced in rats with induced ocular hypertension relative to control retinas^3, 37^. PNU-282987 (5 μL, 100 μM) was injected intravitreally at 0, 1, and 2 weeks after the induction of ocular hypertension by EVC. The dose used in our study was selected based on the results of a previous study in which glaucoma was induced *in vivo* by injection of hypertonic saline into the episcleral veins^38^. RGC survival was evaluated 1 week after the third dose (i.e., 3 weeks after EVC). Representative images of flat-mounted retinas and 20× magnified images obtained 3 weeks after the induction of ocular hypertension are shown in Fig. 2A–D. The RGC densities were assessed in two regions of each retinal quadrant (dorsal, nasal, temporal, and ventral): a central region 1 mm from the optic nerve head and a peripheral region 3 mm from the optic nerve head (Fig. 2E). The density of FluoroGold-positive RGCs was significantly greater in ocular hypertensive eyes treated with PNU-282987 than that in ocular hypertensive eyes treated with or without the vehicle. As shown in Fig. 2F, the mean densities of RGCs in the central (*n* = 10, *p* = 0.010) and peripheral (*n* = 10, *p* = 0.001) regions were 4,510 ± 60 cells/mm^2^ and 2,800 ± 112 cells/mm^2^ (mean ± SE), respectively, in control eyes vs. 3,828 ± 185 cells/mm^2^ and 1,927 ± 110 cells/mm^2^, respectively, in eyes with EVC-induced ocular hypertension. In eyes with ocular hypertension, PNU-282987 significantly enhanced RGC survival in the central and peripheral regions, resulting in mean RGC densities of 4,282 ± 62 cells/mm^2^ and 2,572 ± 93 cells/mm^2^, respectively (*n* = 10) compared with 3,833 ± 118 cells/mm^2^ (*p* = 0.013) and 1,985 ± 64 cells/mm^2^ (*p* = 0.001), respectively, in vehicle-treated eyes (*n* = 10). These data indicate that the activation of α7-nAChR significantly enhanced the survival of RGCs in eyes with ocular hypertension.

**Figure 3:**
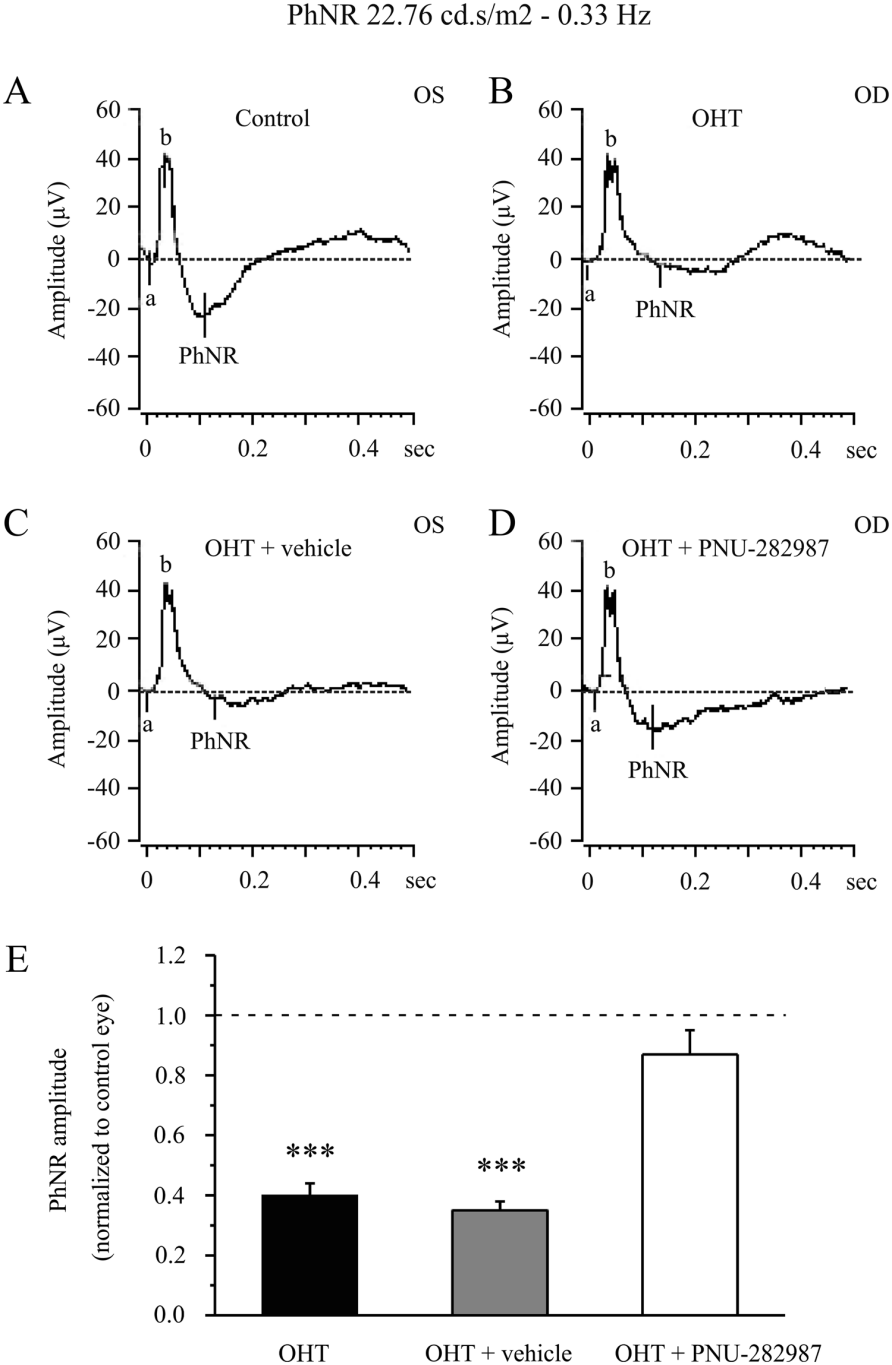
PhNR of normal and glaucomatous rat eyes. (**A,B**) Representative traces of the “a” wave, the “b” wave, and PhNR in a control eye (OS) and a glaucomatous eye (OD) at step 3 with the stimulus applied at 22.76 cd.s/m^2^–0.33 Hz. (**C,D**) Representative waves in a vehicle-treated glaucomatous eye (OS) and a PNU-282987-treated glaucomatous eye (OD) at the same step and stimulus used in (**A,B**). (**E**) Quantitative analysis of PhNR amplitude (*n* = 8). The amplitude was normalized to the amplitude in control retinas. ***p < 0.001 (one-way analysis of variance). The results in **E** are expressed as the mean ± SE. a, “a” wave; b, “b” wave; OHT, ocular hypertension; PhNR, photopic negative response.

We next tested whether the effects of PNU-292987 on RGC survival are associated with retinal function by performing electroretinography (ERG). For this purpose, we analyzed the photopic negative response (PhNR), a sensitive marker of inner retinal function that is damaged in glaucoma patients^39, 40^. The amplitude of the PhNR is proportional to the number of functional RGCs^39–43^. As shown in Fig. 3, at 3 weeks after EVC, the amplitude of the PhNR was reduced in hypertensive eyes treated without (40% ± 4% (mean ± SE), *p* = 0.001, *n* = 8; Fig. 3B,E) or with the vehicle (35% ± 3%, *p* = 0.001, *n* = 8; Fig. 3C,E) relative to control eyes (Fig. 3A,E). Administration of PNU-282987 increased the PhNR amplitude in hypertensive eyes to 87% ± 8% (Fig. 3D,E) of that in control eyes (Fig. 3A,E). These results confirm that intraocular hypertension leads to serious deficits in RGC function and indicate that these deficits are prevented by administration of the α7-nAChR agonist PNU-282987.

### Chronic glaucoma alters mIPSC kinetics and GAD65/67 and GABA protein expression, and these changes are prevented by PNU-282987

**Figure 4:**
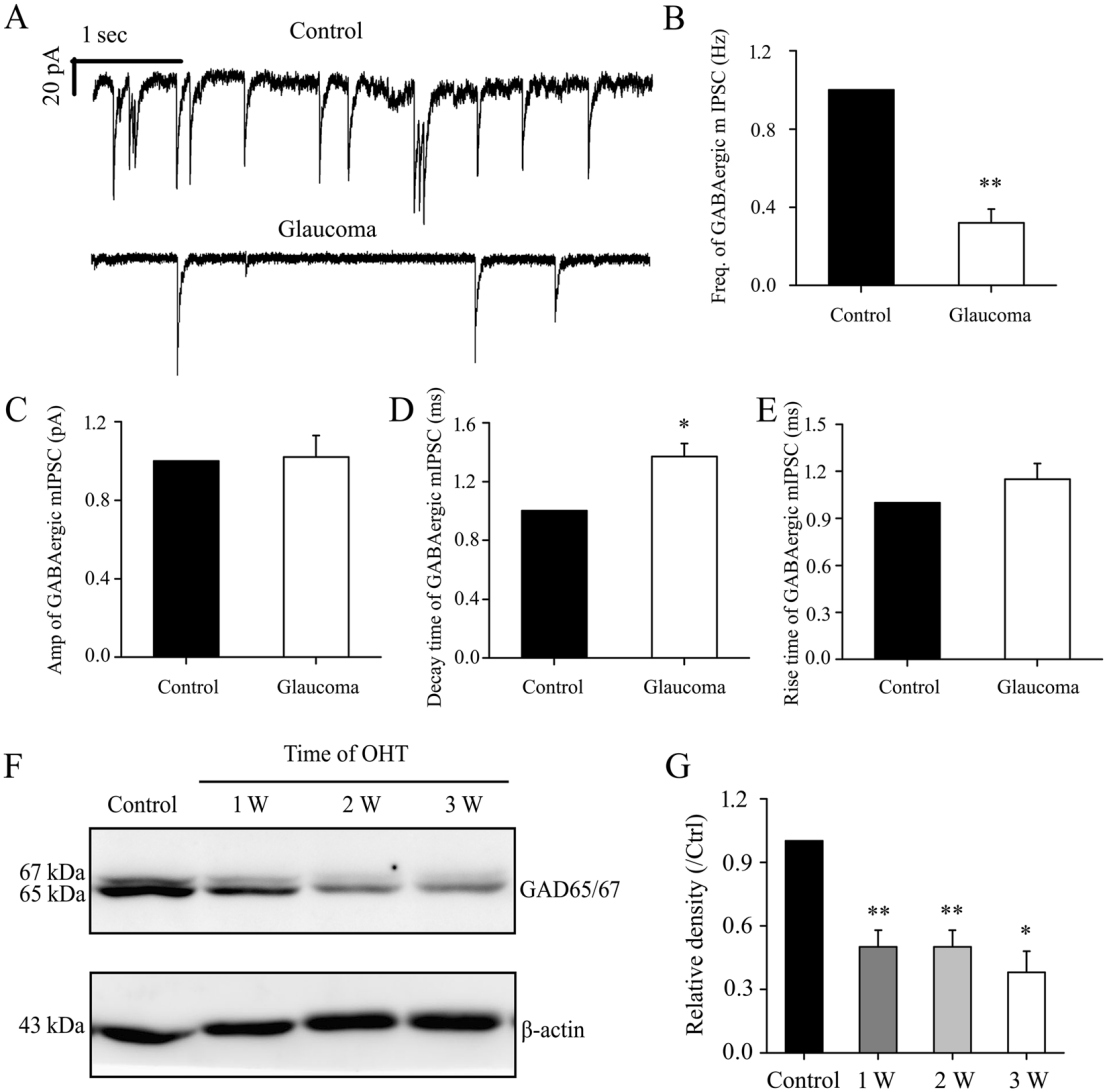
Effects of chronic glaucoma on retinal mIPSCs and GAD65/67 protein expression. (**A**) Representative traces of voltage clamp recordings of GABAergic mIPSCs in the presence of TTX (1 μM) in control and glaucomatous retinas. The baseline frequency of the mIPSCs is markedly reduced in the glaucomatous retina compared with the control retina. (**B–E**) Histograms showing the mean frequency, amplitude, decay time, and rise time of the mIPSCs for control and glaucomatous retinas. The mIPSC frequency (decreased by 68%, *n* = 4, **p < 0.01) and decay time (increased by 37%, *n* = 5, *p < 0.05) are significantly different between the control and glaucomatous retinas. (**F**) Western blotting of GAD65/67 protein expression in glaucomatous and control retinal homogenates. The full-length blots are presented in Supplementary Figure S1. (**G**) Densitometric analysis of GAD65/67, confirming the downregulation of GAD65/67 protein expression in the glaucomatous retina at 1 week (*n* = 4), 2 weeks (*n* = 4), and 3 weeks (*n* = 3) after inducing ocular hypertension. *p < 0.05 and **p < 0.01 (one-way analysis of variance). GAD65/67 expression was normalized to β-actin level and is shown relative to its expression in control retinas. The results in **B–E** and **G** are expressed as the mean ± SE. GAD, glutamic acid decarboxylase; OHT, ocular hypertension; w, week.

We next performed whole-cell patch-clamp studies to examine whether the kinetics of mIPSC in RGCs were altered in glaucomatous retinas. The mIPSCs were recorded using electrodes filled with CsCl solution (electrode impedance 4–8 MΩ) containing tetrodotoxin (TTX; 1 μM), 6-cyano-7-nitroquinoxaline-2,3-dione (CNQX; 10 μM), D-2-amino-5-phosphonovalerate (AP5; 50 μM), and strychnine (5 μM). The location of RGCs in retinal slices was determined by infrared differential interference contrast (IR-DIC) microscopy using a water-immersion objective lens (magnification 40×) and intracellular injection of Lucifer Yellow^44^. Individual RGCs were clamped at a holding potential of −70 mV. Figure 4A shows traces of GABAergic mIPSCs from RGCs in control and glaucomatous retinal slices. The mIPSC frequency (mean ± SE) was 2.79 ± 0.63 Hz for control RGCs and 0.78 ± 0.11 Hz in glaucomatous RGCs (*n* = 4, *p* = 0.040), a reduction to 32% ± 7% of the control level (*n* = 4, *p* = 0.002; Fig. 4B). However, the mean mIPSC amplitude in control and glaucomatous RGCs was not significantly different (15.26 ± 1.17 pA in control RGCs vs. 15.08 ± 1.01 pA in glaucomatous RGCs; *n* = 5, *p* = 0.919), equivalent to 102% ± 11% of the control level (*n* = 5, *p* = 0.882; Fig. 4C). A significant increase in the mIPSC decay time was observed in glaucomatous RGCs (control vs. glaucomatous RGCs: 10.56 ± 0.75 ms vs. 14.40 ± 1.19 ms; *n* = 5, *p* = 0.017), representing an increase to 137% ± 9% of the control level (*n* = 5, *p* = 0.017; Fig. 4D). The mIPSC rise time did not differ significantly between control and glaucomatous RGCs (5.13 ± 0.61 ms vs. 5.84 ± 0.80 ms, *n* = 4, *p* = 0.271; 115% ± 10% of the control level, *n* = 4, *p* = 0.246; Fig. 4E).

**Figure 5:**
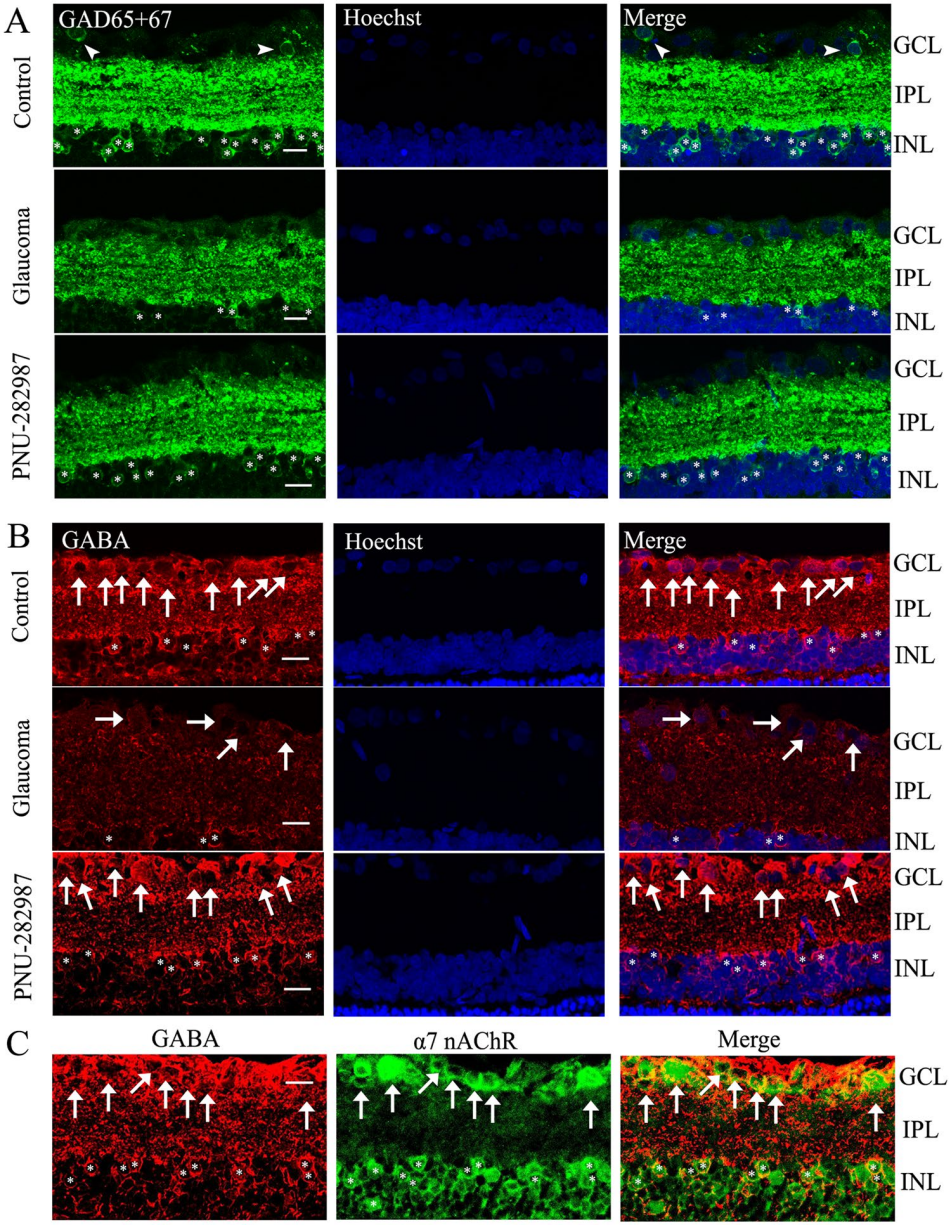
PNU-282987 prevents reductions in GAD65/67 and GABA immunoreactivity caused by chronic ocular hypertension. (**A,B**) Micrographs of 10 μm thick transverse sections of retinas from control and glaucomatous rats. (**A**) Compared with control retina (top panels in A), GAD65/67 immunoreactivity is reduced in glaucomatous retina (middle panels in A), corresponding to reduced expression in the INL (asterisks), inner plexiform layer (IPL), and GCL (arrowheads) of the glaucomatous retina. These effects of ocular hypertension are prevented by administration of PNU-282987 (bottom panels in A). (**B**) GABA immunohistochemistry is decreased in the glaucomatous retina (middle panels in B, arrows, asterisks) relative to controls (top panels in B, arrows, asterisks). PNU-282987 (bottom panels in B, arrows, asterisks) prevents the effects of ocular hypertension on GABA expression. (**C**) Double-label immunohistochemistry shows that GABA (red) is colocalized with α7-nAChRs (green). Scale bar, 15 μm. INL, inner nuclear layer; IPL, inner plexiform layer; GCL, ganglion cell layer. The arrowheads indicate sparse amacrine cells, arrows indicate RGCs, and asterisks indicate amacrine cells.

Next, we determined whether the electrophysiological characteristics of the cells were associated with changes in the GABAergic system. Using western blotting, we found that the expression of GAD65/67, a marker of GABA synthesis, was significantly decreased in glaucomatous retinas (Fig. 4F). GAD65/67 protein expression in glaucomatous retinas decreased to 50% ± 8% at 1 week (*n* = 4, *p* = 0.007), 50% ± 8% at 2 weeks (*n* = 4, *p* = 0.008), and 38% ± 10% at 3 weeks (*n* = 3, *p* = 0.026) relative to the control retinas (Fig. 4G). Using immunohistochemistry, we also found that the density of GAD65/67 was significantly decreased in glaucomatous retinas relative to the control retinas (Fig. 5A). Consistent with the observed changes in GAD65/67, GABA immunoreactivity was also significantly reduced in glaucomatous retinas (Fig. 5B). The absence of GAD65/67 may have contributed to the lower mIPSC frequency observerd in glaucomatous RGCs.

**Figure 6:**
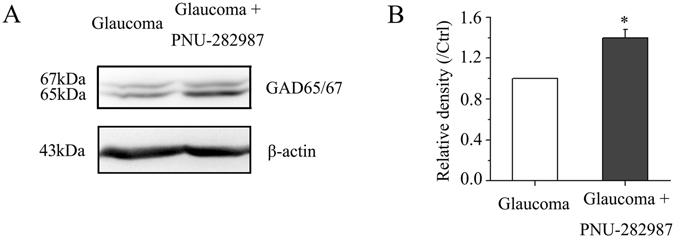
PNU-282987 prevents the reduction in GAD65/67 protein expression caused by chronic ocular hypertension. (**A**) Western blotting showing the effects of PNU-282987 on GAD65/67 protein expression in glaucomatous retina. The full-length blots are presented in Supplementary Figure S1. (**B**) Densitometric analysis of GAD65/67 confirming that PNU-282987 upregulates GAD65/67 protein expression in glaucomatous retina (*n* = 6). *p < 0.05 (Student’s paired *t* test). GAD65/67 expression was normalized to β-actin and is shown relative to its expression in control retinas. The results in **B** are expressed as the mean ± SE. GAD, glutamic acid decarboxylase.

To test whether activation of the α7-nAChR is required for the observed increases in GAD65/67 and GABA expression, PNU-282987 was injected intravitreally on day 0 (the day of EVC) and every 7 days thereafter following EVC. Immunofluorescence and western blotting were performed 3 weeks after EVC. The results of immunofluorescence showed that administration of PNU-282987 increased GAD65/67 and GABA expression levels in RGCs (Fig. 5A,B). Double-label immunofluorescence revealed that α7-nAChR and GABA were colocalized in the INL and GCL (Fig. 5C). Figure 6A shows the representative western blotting results of the expression levels of GAD65/67 after PNU-282987 (10 µM) treatment for 3 weeks. PNU-282987 increased GAD65/67 protein expression in glaucomatous retinas to 40% ± 8% of that in vehicle-treated retinas (*n* = 6, *p* = 0.028; Fig. 6B). We found that low α7-nAChR expression in glaucomatous retinas was often accompanied by low GAD65/67 and GABA expression levels. These findings indicate that the loss of α7-nAChR is associated with decreased GABA synthesis and reduced GABAergic synaptic input to RGCs.

### PNU-282987 increases the GABAergic mIPSCs of RGCs

**Figure 7:**
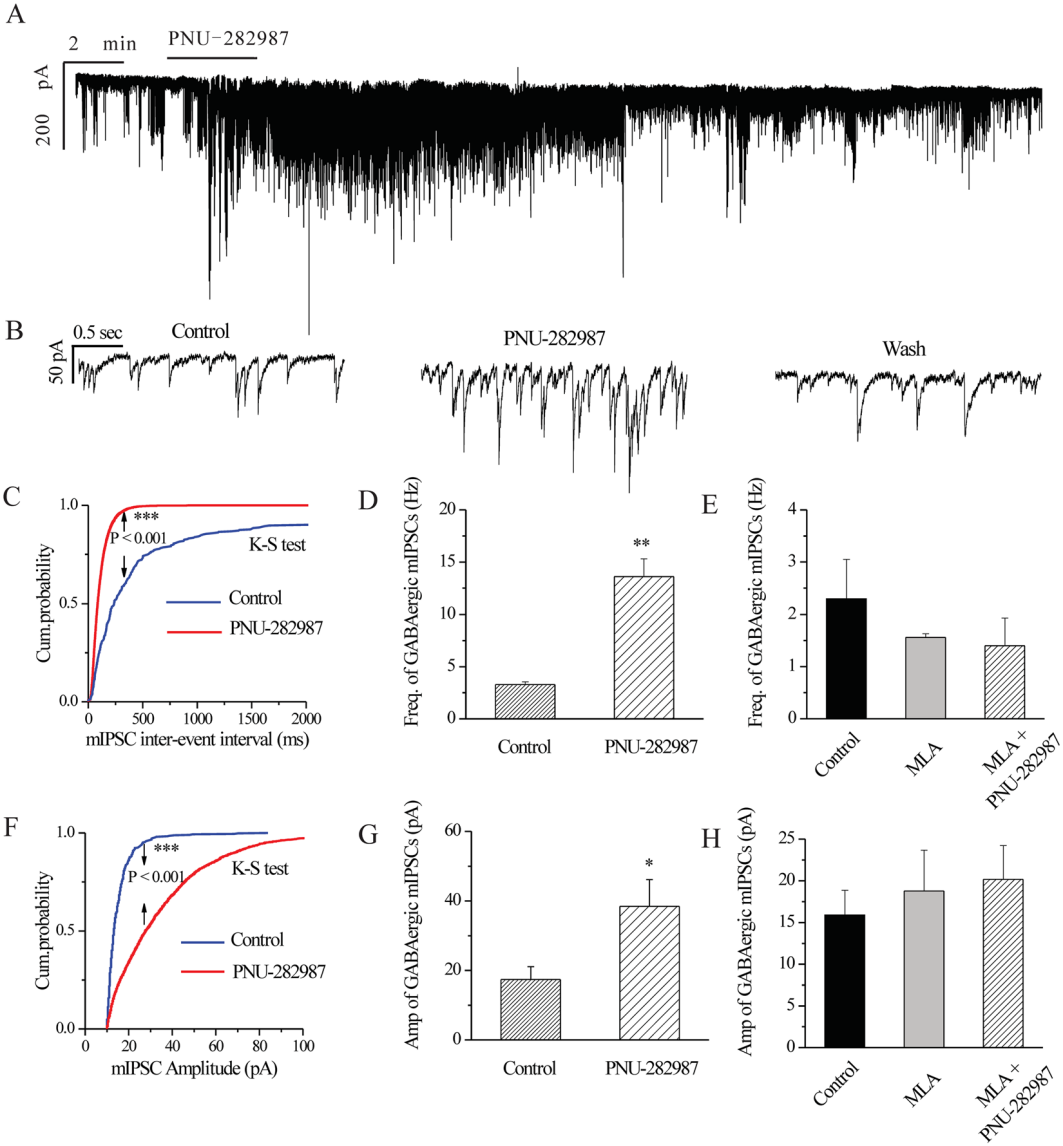
PNU-282987, a selective α7-nAChR agonist, increases the frequency and amplitude of mIPSCs in control rat RGCs. (**A**) Voltage clamp recording (at −70 mV) of a representative normalized RGC shows that PNU-282987 increases the frequency and amplitude of GABAergic mIPSCs in RGCs. Vertical bar, 200 pA; horizontal bar, 2 min. (**B**) The recording of the RGC on an expanded scale in time, vertical calibration bar, 50 pA; horizontal calibration bar, 0.5 s. (**C,F**) Cumulative inter-event interval and amplitude distributions of the mIPSCs of a representative RGC before and during PNU-282987 application. PNU-282987 causes a leftward shift in the inter-event interval (**C**) and a rightward shift in the amplitude (**F**) distribution curves, indicating that PNU-282987 significantly increases mIPSC frequency and amplitude (*n* = 9). ***p < 0.001 (Kolmogorov**–**Smirnov test). (**D,G**) Quantification of the frequency (**D**) and amplitude (**G**) of the mIPSCs (*n* = 5). (**E,H**) Pre-incubation with MLA (1 μM), a selective α7-nAChR antagonist, inhibits the effects of PNU-282987 (10 μM) on the frequency (**E**) and amplitude (**H**) of mIPSCs (*n* = 3). Administration of MLA alone does not affect the baseline frequency (**E**) or amplitude (**H**) of the mIPSCs. *p < 0.05, **p < 0.01 (Student’s paired *t* test). The results in **D**,**E**,**G**, and **H** are expressed as the mean ± SE.

We next investigated the synaptic mechanism underlying the neuroprotective effects of PNU-282987. In normal RGCs, exposure to PNU-282987 (10 μM) significantly increased the frequency and amplitude of the GABAergic mIPSCs of RGCs after preincubation with 1 μM TTX (Fig. 7A,B). The effects of PNU-282987 on the cumulative distributions of the inter-event intervals and the amplitudes of GABAergic mIPSCs, as assessed by the Kolmogorov–Smirnov test, are shown in Fig. 7C (*n* = 9, *p* = 0.001) and Fig. 7F (*n* = 9, *p* = 0.001), respectively. PNU-282987 reduced the inter-event interval and increased the amplitude of mIPSCs compared with the control. The frequency of the GABAergic mIPSCs was increased from 3.28 ± 0.27 to 13.6 ± 1.71 Hz (*n* = 5, *p* = 0.003; Fig. 7D). The amplitude of the GABAergic mIPSCs was increased from 17.39 ± 3.65 to 38.46 ± 7.69 pA (*n* = 5, *p* = 0.012; Fig. 7G). The PNU-282987-induced responses began within 3–4 min of application of the drug and were reversible by washout of PNU-282987. The effects of PNU-282987 on the frequency and amplitude of the GABAergic mIPSCs were blocked by a highly selective α7-nAChR antagonist, methyllycaconitine (MLA) (Fig. 7E,H). MLA alone did not significantly affect the baseline frequency or amplitude of the GABAergic mIPSCs (Fig. 7E,H). At the end of the experiments, application of the selective GABA_A_ receptor antagonist SR95531 (10 μM) abolished all of the mIPSCs.

**Figure 8:**
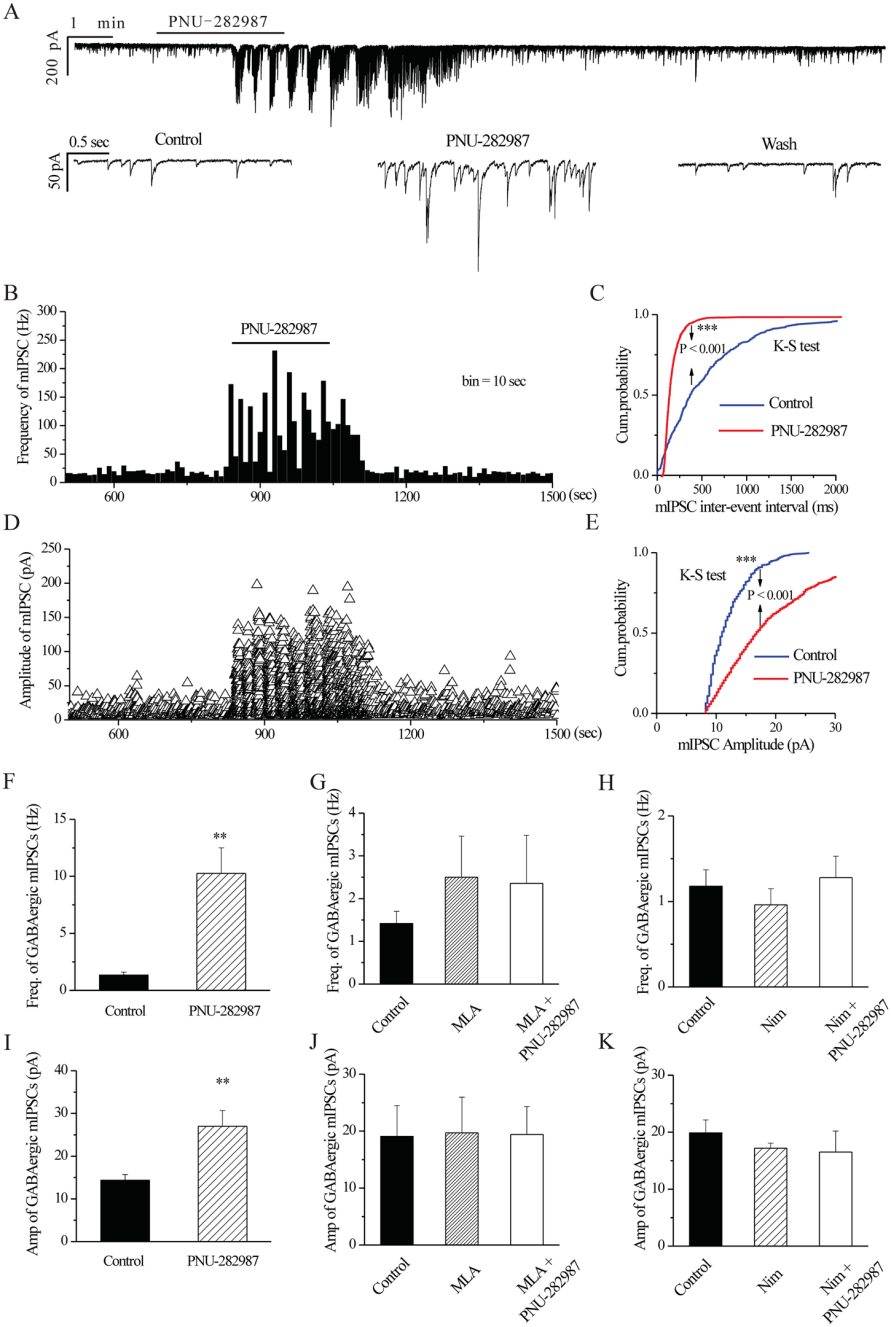
The effects of PNU-282987 on the frequency and amplitude of mIPSCs in glaucomatous RGCs are prevented by MLA or nimodipine. (**A**) Continuous trace of a representative experiment showing mIPSCs at baseline, during PNU-282987 application (10 μM), and during recovery. The trace is shown at a magnified scale in the lower panel in A. Vertical bar, 200 pA (top) or 50 pA (bottom); horizontal bar, 1 min (top) or 0.5 s (bottom). (**B,D**) Frequency (10 s bin) and amplitude histograms of the mIPSCs of the trace in **A**, showing the effects of PNU-282987. (**C,E**) Cumulative inter-event interval and amplitude distribution of the mIPSCs of a representative neuron during control recording and during PNU-282987 application. PNU-282987 significantly shifts the distribution of inter-event intervals to the left (**C**) and the distribution of mIPSC amplitudes (**E**) to the right. The PNU-282987**-**induced changes in the distribution of inter-event intervals and amplitudes are statistically significant (*n* = 6). ***p < 0.001 (Kolmogorov**–**Smirnov test). (**F,I**) Quantification of the frequency (**F**, *n* = 11) and amplitude (**I**, *n* = 10) of the mIPSCs. (**G,H,J,K**) Pretreatment with MLA (**G,J**; 1 μM) or nimodipine (**H,K**; 10 μM) prevents the effects of PNU-282987 on the frequency and amplitude of mIPSCs (*n* = 3). Pretreatment with MLA (**G,J**) or nimodipine (**H,K**) does not affect the baseline frequency or amplitude of the mIPSCs. **p < 0.01 vs. control (Student’s paired *t* test). The results in **F–K** are expressed as the mean ± SE.

Similar changes were observed in glaucomatous retinas, where PNU-282987 (10 μM) also significantly increased the frequency and amplitude of GABAergic mIPSCs (Fig. 8A). Frequency histogram (Fig. 8B) and running amplitude (Fig. 8D) of GABAergic mIPSCs in a representative RGC, which showed the time couse of the frequency and amplitude response to PNU-282987 application. The effects of PNU-282987 on the cumulative distributions of the inter-event intervals and amplitudes of GABAergic mIPSCs, as assessed by the Kolmogorov–Smirnov test, are shown in Fig. 8C (*n* = 6, *p* = 0.001) and Fig. 8E (*n* = 6, *p* = 0.001), respectively. PNU-282987 markedly decreased the inter-event interval and increased the amplitude of mIPSCs relative to the control. The frequency of GABAergic mIPSCs (mean ± SE) was 1.35 ± 0.25 Hz before PNU-282987 application and increased to 10.26 ± 2.23 Hz in the presence of PNU-282987 (*n* = 11, *p* = 0.002; Fig. 8F). The amplitude of the GABAergic mIPSCs increased from 14.36 ± 1.36 pA before PNU-282987 application to 27 ± 3.69 pA during PNU-282987 application (*n* = 10, *p* = 0.003; Fig. 8I). These responses occurred within 3–4 min and were reversible by the washout of PNU-282987. At the end of the experiments, 10 μM SR95531 abolished all GABAergic mIPSCs in the RGCs. In cells preincubated with MLA (100 nM), the addition of 10 μM PNU-282987 did not elicit significant changes in the frequency or amplitude of GABAergic mIPSCs (Fig. 8G,J). In addition, the effects of PNU-282987 were suppressed by the L-type Ca^2+^ channel antagonist nimodipine (Fig. 8H,K). These results indicate that α7-nAChR-induced GABA release may be triggered by Ca^2+^ entry into synaptosomes through L-type voltage-dependent Ca^2+^ channels (VDCC).

### The protective effects of PNU-282987 on RGCs are blocked by the selective GABA_A_ receptor antagonist SR95531

**Figure 9:**
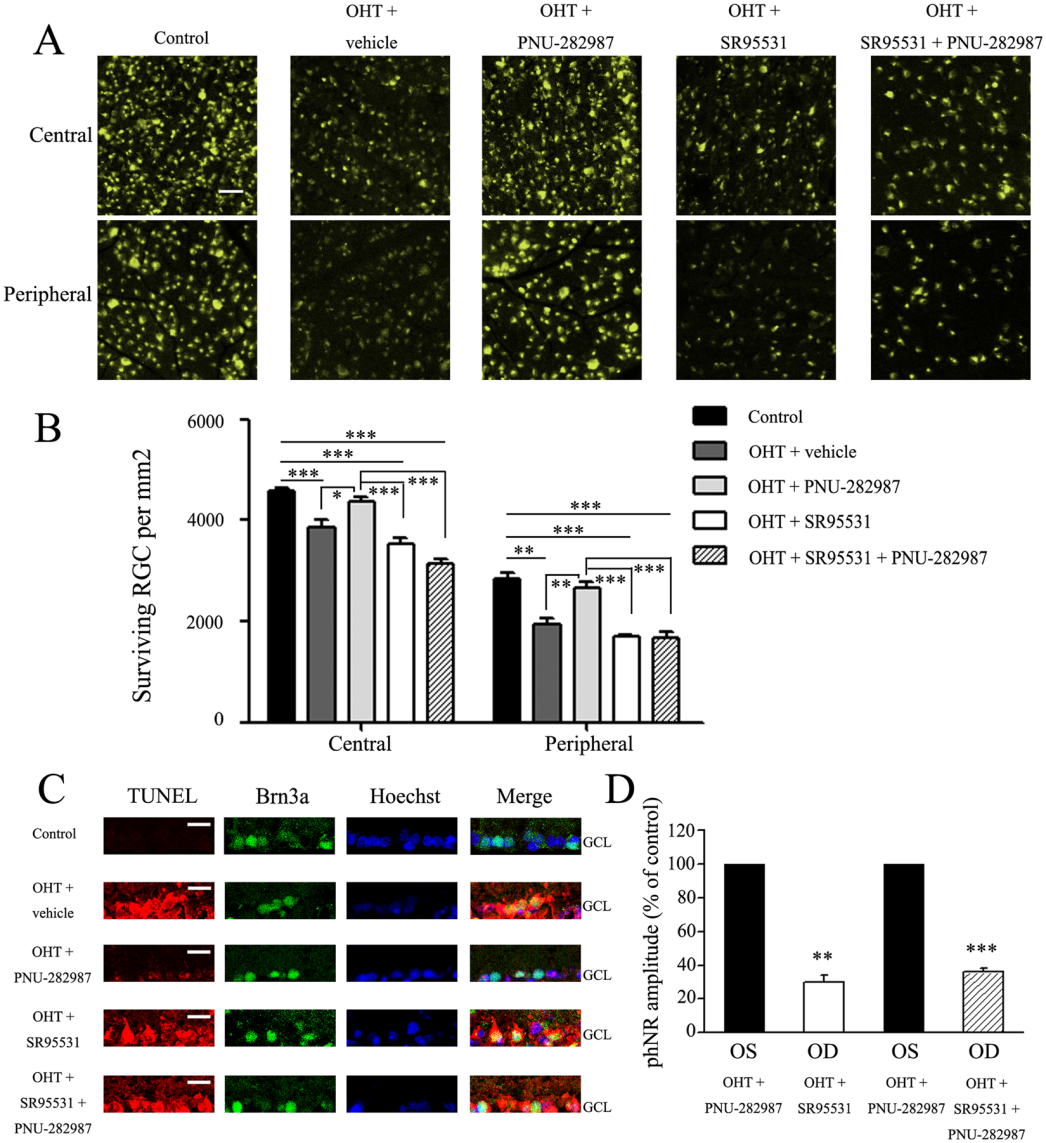
The effects of PNU-282987 *in vitro* and *in vivo* are blocked by intravitreal administration of SR95531, a selective GABA_A_ receptor antagonist. (**A**) FluoroGold labeling of surviving RGCs in the central (top panel) and peripheral (bottom panel) regions of retina. The images were obtained at high magnification (scale bar, 50 μm). Administration of SR95531 decreases the effects of PNU-282987 on RGC viability. (**B**) Quantification of RGC survival in control and glaucomatous retinas following treatment with the vehicle, PNU-282987, SR95531, and SR95531 + PNU-282987 (*n* = 7). *p < 0.05, **p < 0.01, ***p < 0.001 (one-way analysis of variance). (**C**) Representative TUNEL (red), Brn3a (green), Hoechst (blue), and merged confocal microscopic images of the ganglion cell layer. All images were obtained at the same magnification. Scale bars, 20 μm. (**D**) Quantification of the PhNR amplitude in glaucomatous retinas of eyes treated with SR95531 or SR95531 + PNU-282987 relative to eyes treated with PNU-282987. **p < 0.01, ***p < 0.001 (Student’s paired *t* test). The results in B and D are expressed as the mean ± SE. OHT, ocular hypertension.

The vehicle, PNU-282987, SR95531 or SR95531 + PNU-282987 was intravitreally injected at 0, 1, and 2 weeks after induction of ocular hypertension by EVC. RGC survival was evaluated 1 week after the third dose of the studied drugs (i.e., 3 weeks after EVC). Representative images of the experimental retinas (magnification 20×) at 3 weeks after EVC are shown in Fig. 9A. As illustrated in Fig. 9B, the density of RGCs (mean ± SE) in SR95531-treated hypertensive eyes was similar to that in vehicle-treated hypertensive eyes in both the central (3,541 ± 100 vs. 3,860 ± 155 cells/mm^2^, *n* = 7, *p* = 0.152) and peripheral (1,688 ± 47 vs. 1,952 ± 115 cells/mm^2^, *n* = 7, *p* = 0.117) regions, indicating that SR95531 does not exacerbate the effects of ocular hypertension (Fig. 9A,B). Treatment with PNU-282987 was associated with better RGC survival than treatment with SR95531 in both the central (4,370 ± 96 vs. 3,541 ± 100 cells/mm^2^, *n* = 7, *p* = 0.001) and peripheral (2,663 ± 111 vs. 1,688 ± 47 cells/mm^2^, *n* = 7, *p* = 0.001) regions (Fig. 9A,B). To confirm that SR95531 blocked the effects of PNU-282987, ocular hypertensive eyes were injected intravitreally with SR95531 (5 μL, 100 μM) followed by PNU-282987 (100 μM). Pretreatment with SR95531 prevented the effect of PNU-282987 on RGC survival, resulting in densities (mean ± SE) of 3,134 ± 103 cells/mm^2^ in the central region (*n* = 7, *p* = 0.121; Fig. 9A,B) and 1,672 ± 105 cells/mm^2^ in the peripheral region (*n* = 7, *p* = 0.808; Fig. 9A,B).

Next, we used terminal deoxynucleotidyl transferase dUTP nick end labeling (TUNEL) staining to detect apoptotic RGCs in the experimental retinas. As shown in Fig. 9C, the TUNEL-positive signal overlapped with the Brn3a (the RGC marker) and the Hoechst images, thereby demonstrating apoptosis of RGCs. No TUNEL-positive cells were detected in the control retinas, and PNU-282987 significantly reduced the number of TUNEL-positive cells in hypertensive eyes relative to the vehicle and SR95531 treatments. However, pretreatment with SR95531 (100 μM) prevented the attenuating effect of PNU-282987 on the number of TUNEL-positive cells. Consistent with these findings, ERG showed that the amplitude of PhNR in glaucomatous retinas was decreased by SR95531 to 30% ± 4% of that of the PNU-282987-treated groups (*p* = 0.001, *n* = 5; Fig. 9D). SR95531 also suppressed the effects of PNU-282987 on PhNR, which decreased to 36% ± 2% of that of the PNU-282987-treated group in eyes treated with SR95531 followed by PNU-282987 (*p* = 0.001, *n* = 5; Fig. 9D). These results indicate that the protective effects of PNU-282987 with respect to RGC density, RGC function, and number of TUNEL-positive cells in glaucomatous eyes were blocked by SR95531.

## Discussion

**Figure 10:**
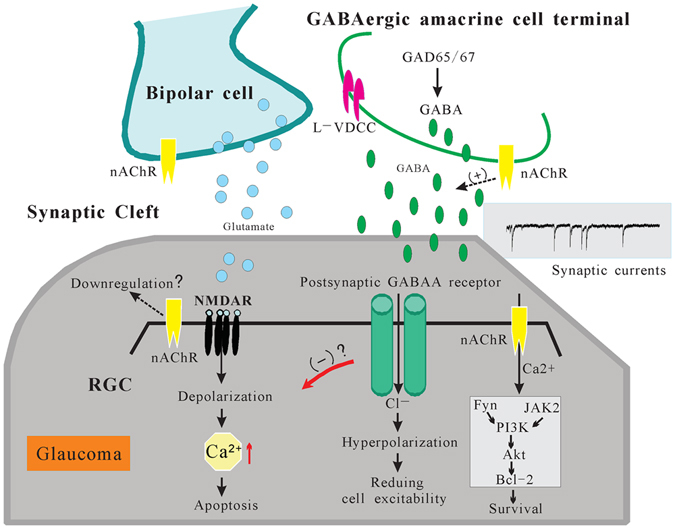
Schematic model showing possible mechanisms for the neuroprotective effects of α7-nAChR activation. Excessive activation of postsynaptic NMDA receptors produces a sustained depolarizing influx of Ca^2+^ in RGCs that eventually leads to neurodegeneration. In our study, the activation of presynaptic α7-nAChR by PNU-282987 increased GABA release from presynaptic GABAergic amacrine cells. Thus, the activation of GABA_A_ receptors can attenuate the activity of excitatory NMDARs. RGC, retinal ganglion cell; nAChR, nicotinic acetylcholine receptor; GAD, glutamic acid decarboxylase; L-VDCC, L-type voltage-dependent Ca^2+^ channels.

There are currently no effective neuroprotectants for the treatment of glaucoma. Herein, for the first time, we report that PNU-282987 improves RGC survival and function in an animal model of chronic ocular hypertension. First, we showed that intraocular hypertension rapidly downregulates α7-nAChR mRNA and protein expression in RGCs. Second, we demonstrated that the administration of PNU-282987 protects the somas of RGCs and prevents the decline in RGC function. The protective effects of PNU-282987 were blocked by the selective GABA_A_ receptor antagonist SR95531. Third, whole-cell patch-clamp recordings revealed decreases in the baseline frequency and increases in the decay time of mIPSCs of RGCs in glaucomatous retinas. Fourth, we provided evidence that deficits in α7-nAChR expression are accompanied by downregulation of GAD65/67 and GABA. Finally, we showed that PNU-282987 significantly increases the frequency and amplitude of the GABAergic mIPSCs of RGCs via a mechanism involving L-type VDCCs. The effects of PNU-282987 at the synaptic level were completely prevented by MLA. Altogether, the results of this study reveal a novel mechanism by which activation of α7-nAChRs modulates the neuronal GABAergic system and thereby helps protect RGCs. A schematic model of possible mechanisms is presented in Fig. 10.

Alpha7-nAChRs are highly permeable to Ca^2+^ ^45^. However, both neurotoxic and neuroprotective roles of Ca^2+^ have been reported^46^. Brandt *et al*. suggested that Ca^2+^ is neuroprotective against glutamate-induced cellular damage at low concentrations but has pro-apoptotic effects at higher concentrations^9^. The finding that rapid desensitization of α7-nAChRs may limit cellular Ca^2+^ entry^47, 48^ implies that α7-nAChRs act as tonic modulators of intracellular events such as signal transduction and neurotransmitter release. Great loss of nAChRs occurs in neurodegenerative diseases such as Alzheimer’s disease^49^. We are the first to show that α7-nAChR expression is downregulated in a rat model of chronic glaucoma. The capacity of α7-nAChR agonists to protect RGCs and improve their function was confirmed by FluoroGold staining and ERG.

The common pathogenic mechanisms underlying neuronal cell death include abnormal glutamate outflow and decompensated inhibitory mechanisms, resulting in the loss of cellular homeostasis. Ischemic insults in the brain reduce endogenous GABA synthesis and release. Administration of the GABA_A_ receptor agonist, muscimol, the GABA_A_ mimetic agent, clomethiazole, and the GABA uptake inhibitor, CI-966, have been shown to be beneficial in experimental models of stroke^26^. Preoperative oral gabapentin elicits a modest decrease in IOP in some elderly patients undergoing elective intraocular surgery, an effect that may be mediated by elevated GABA concentrations^50^.

GABA is synthesized by the GAD65 and GAD67 isoforms of the rate-limiting enzyme GAD. GAD65 is expressed at GABAergic synapses and GAD67 is expressed in the soma^34^. GAD plays an essential role in maintaining a balance between excitation and inhibition in the CNS, and a reduction in GAD expression may cause a decrease in GABA production. In the current study, using a rat model of chronic glaucoma, we presented evidence that glaucomatous neuropathy may involve a decrease in the GABAergic presynaptic activity of RGCs^27, 51^. The reduced retinal GAD65/67 and GABA levels and the decreased GABAergic mIPSC frequencies observed in the glaucomatous retinas in this study are indicative of deficits in retinal GABA synthesis and a conspicuous reduction in GABA release. In comparison, Quigley *et al*. reported that amacrine cells identified by GABA labeling were not affected in glaucoma induced by translimbal trabecular laser treatment^29^. The reason for this discrepancy is unclear, but it is important to consider that the experiments of Quigley *et al*. were conducted 1–3 months after laser treatment, whereas we observed changes in GABAergic immunoreactivity and mIPSC kinetics within 1–3 weeks after inducing ocular hypertension. Another possible explanation for the differences in the results is the differences in the experimental models of glaucoma used in the two studies.

It is also worth noting that PNU-282987 increased GAD65/67 expression, and this increase was accompanied by increases in GABA activity and in the frequencies and amplitudes of the GABAergic mIPSCs of RGCs. Therefore, it is tempting to speculate that a cause-and-effect relationship exists between the downregulation of α7-nAChRs and the observed changes in the GABAergic system. Deletion of the α7-nAChR gene in mice alters the balance between excitatory and inhibitory inputs and impairs cortical GABAergic neuronal development in models of schizophrenia^34^. The ratio between excitatory input and inhibitory input is a fundamental feature of neural networks, and endogenous nicotinic cholinergic signaling plays an important role in network construction^52^.

Our data show that an α7-nAChR agonist (PNU-282987) increases the frequency of GABAergic mIPSCs of RGCs, suggesting that it enhances the presynaptic release of GABA onto RGCs. Previous studies have shown that nicotine increases the GABAergic input of rat dorsal raphe serotonergic neurons by activating α7-nAChRs^53^. Yang *et al*. reported that α6β2-nAChRs are located on presynaptic GABAergic boutons within the ventral tegmental area and that they increase GABA release onto dopaminergic neurons^54^. However, another study suggested that co-activation of presynaptic endocannabinoid and muscarinic acetylcholine receptors (mAChRs) reduces GABA release^55^. In contrast, we showed that an α7-nAChR agonist increased the presynaptic release of GABA onto RGCs. Moreover, α7-nAChR-mediated GABA release was not inhibited by pretreatment with TTX, indicating that the observed effects are not dependent on the activity of Na^+^ channels. Our results also suggest that α7-nAChRs are located on the terminals of the GABAergic amacrine neurons that innervate RGCs.

It is also notable that α7-nAChR-evoked, TTX-independent GABA release is Ca^2+^-dependent and is blocked by nimodipine. This finding indicates that Ca^2+^-inflow-associated membrane depolarization leads to the opening of L-type VDCCs^53^. Accordingly, in RGCs, the stimulation of α7-nAChRs located on GABAergic amacrine terminals causes changes in synaptic transmission via a mechanism involving VDCC activation. However, the PNU-282987-induced increases in the amplitude of GABAergic mIPSCs of RGCs were prevented by the L-type VDCC blocker nimodipine, suggesting that the observed increase in mIPSCs amplitude is unlikely to be a postsynaptic effect. Instead, it is more likely caused by presynaptic effects of α7-nAChRs, in particular by increased summation of synaptic events and recruitment of additional presynaptic neurons. The greater increase in the frequency of GABAergic mIPSCs during exposure to PNU-282987 (76%) may have been sufficient to generate the increased mIPSC amplitude^56^. However, additional studies in which GABA-induced currents and the activity of GABA receptors are examined are needed to confirm whether postsynaptic effects of α7-nAChRs in RGCs are involved.

In conclusion, the present study reveals a crucial role of α7-nAChRs in glaucomatous neurodegeneration of the retina based on the results of multiple complementary experiments. Importantly, our results confirm that the downregulation of α7-nAChRs affects the balance between excitatory and inhibitory inputs in the retina. We also demonstrated the presence of a novel synaptic mechanism underlying the neuroprotective roles of α7-nAChR that involves excitatory GABAergic presynaptic activity. Accordingly, it is possible that the use of α7-nAChR agonists could improve the neural equilibrium in the glaucomatous retina. These findings contribute to our understanding of the role of synapses in RGC injury and may have implications for the development of neuroprotective treatments for glaucoma.

## Materials and Methods

### Animals

All experimental procedures conformed to the ARVO Statement for the Use of Animals in Ophthalmic and Vision Research and the guidelines of Fudan University on the ethical use of animals. Experiments were performed in adult male Wistar rats (200–250 g; age 2 months; SLAC Laboratory Animal Co., Ltd, Shanghai, China). The rats were maintained in an animal facility with a 12 h light/dark cycle at 23 ± 2 °C and a humidity of 60–70%. All efforts were made to minimize the number of animals used and their suffering. The animals were deeply anesthetized by intraperitoneal injection of 10% chloral hydrate (3.6 ml/kg). Proparacaine hydrochloride (0.5% Alcaine; Alcon-Couvreur, Puurs, Belgium) was applied as a topical anesthetic and 0.3% tobramycin (Tobres; Alcon-Couvreur) was applied to prevent post-surgical infection.

### Rat model of ocular hypertension

As previously described^37, 57–59^, three episcleral veins located near the superior and inferior rectus muscles of the right eye were isolated and were precisely cauterized. The contralateral eye underwent a sham operation, which involved isolating the veins without cauterization, as a control. IOP was measured using a calibrated tonometer (Tono-Pen XL; Mentor, Norwell, MA, USA) before surgery and at 30 min, 1 day, 3 days, 5 days, 7 days, 14 days, 21 days, and 28 days after surgery. IOP measurements were recorded as the mean of five consecutive measurements with a deviation of <5%^58^.

### Quantitative PCR

Total RNA was extracted from individual retinas using an RNeasy Plus Mini kit (Qiagen, Venlo, Netherlands). The RNA sample (250 ng) was reverse transcribed into cDNA using the reverse transcription portion of a sensitive qRT-PCR kit (PrimeScript RT reagent kit; Takara Bio Inc, Japan). Real-time PCR was performed using SYBR Premix Ex Taq II (Takara) and the following primers: α7-nAChR: forward, 5′-ACATTGACGTTCGCTGGTTC-3′; reverse, 5′-CTACGGCGCATGGTTACTGT-3′; and glyceraldehyde phosphate dehydrogenase (GAPDH): forward, 5′-ATGGTGAAGGTCGGTGTG-3′; reverse, 5′-GAACTTGCCGTGGGTAGAG-3′. Real-time PCR reactions were performed on a ViiA 7 instrument (Applied Biosystems, Inc., Foster City, CA, USA).

### Western blotting

Western blotting was performed according to our previously described methods^37^. Briefly, retinal lysates were centrifuged at 12,000 × *g* for 10 min at 4 °C. Ten micrograms of each sample was separated by sodium dodecyl sulfate–polyacrylamide gel electrophoresis and electrotransferred onto polyvinyldifluoridine membranes (Immobilon-P; Millipore, Billerica, MA, USA). The membranes were then blocked with 5% nonfat milk for 2 h at room temperature and incubated with the following primary antibodies overnight at 4 °C: goat polyclonal antibody against α7-nAChR (sc-1447, 1:50; Santa Cruz Biotechnology, Santa Cruz, CA, USA) and rabbit polyclonal antibody against GAD65/GAD67 (ab11070, 1:1000; Abcam, Cambridge, MA, USA). The membranes were incubated with horseradish peroxidase-conjugated rabbit anti-goat immunoglobulin (Ig)G (305-035-003, 1:20000; Jackson ImmunoResearch Laboratories, West Grove, PA, USA) and horseradish peroxidase-conjugated goat anti-rabbit IgG (sc-2004, 1:4,000; Santa Cruz Biotechnology). The relative intensities of the protein bands were quantified by scanning densitometry using Image J software (National Institutes of Health, Bethesda, MD, USA). β-actin was used as an internal standard.

### Immunohistochemistry

Immunohistochemistry was performed as described previously^37^. Briefly, 10 μm thick cryosections were fixed in 4% paraformaldehyde for 20 min at room temperature and then incubated in 0.1% Triton X100/phosphate-buffered saline (PBS) for 20 min at 37 °C followed by incubation in 3% bovine serum albumin/PBS for 1 h at room temperature. The cryosections were then incubated with the following primary antibodies for 1 day at 4 °C: rabbit polyclonal antibody against α7-nAChR (sc-5544, 1:20; Santa Cruz Biotechnology), rabbit polyclonal antibody against GAD65/GAD67 (ab11070, 1:200; Abcam), rabbit polyclonal antibody against GABA (A2052, 1:100; Sigma, St. Louis, MO, USA), and goat polyclonal antibody against Brn3a (sc-31984, 1:40; Santa Cruz Biotechnology). The secondary antibodies (all from Invitrogen-Molecular Probes, Eugene, OR, USA) were Alexa Fluor 555-conjugated donkey anti-rabbit IgG antibody (A31572, 1:1,000), Alexa Fluor 488-conjugated goat anti-rabbit IgG antibody (A11070, 1:500), and Alexa Fluor 488-conjugated donkey anti-goat IgG antibody (A11055, 1:500). The sections were finally counterstained with the nucleic acid stain Hoechst 33258 (H3569, 1:2,000; Invitrogen-Molecular Probes) in PBS and imaged using a laser scanning confocal microscope (TCS SP8; Leica Microsystems, Heidelberg, Germany).

### Retrograde labeling of RGCs

Seven days after EVC, anesthetized rats were injected with the fluorescent tracer 3% FluoroGold (2 µL; Sigma) diluted in saline via microinjection into the bilateral superior colliculi (6.0 mm posterior and 2.0 mm lateral to bregma and 4–4.5 mm deep) as previously described^60^. Twenty-one days after EVC (14 days after FluoroGold injection), the retinas were dissected, divided into four quadrants, and flat-mounted on glass slides with the GCL facing up. Twenty images per retina (two from the central and two from the peripheral retina for each quadrant) were captured using a laser scanning confocal microscope (TCS SP8; Leica Microsystems) at a magnification of 20×. The cells were counted by an investigator who was blinded to the study treatments.

### ERG and measurement of the PhNR

Full-field ERG was performed 3 weeks after EVC using an Espion Diagnosys System (Diagnosys LLC, Littleton, MA, USA). Electrical signals were recorded with two 3-mm platinum wire loop electrodes placed on the corneal surfaces of eyes that had been pre-coated with 2.5% hydroxypropyl-methylcellulose solution (Gonak; Akorn, Lake Forest, IL, USA). One subdermal needle electrode inserted into the base of the right leg served as the ground, while the other subdermal needle electrode placed over the nasal bone served as the common reference. Light stimuli were delivered using a ColorDome unit at four different stimulus strengths (11.38 cd.s/m^2^–0.33 Hz, 11.38 cd.s/m^2^–1 Hz, 22.76 cd.s/m^2^–0.33 Hz, and 22.76 cd.s/m^2^–0.33 Hz) in a 4-step test. In each step, the stimulus frequency was 2 Hz, and a green light with an intensity of 10 cd/m^2^ was presented for 4 ms against a green background.

### Preparation of retinal slices

The eyes of rats were rapidly enucleated and transferred to ice-cold, oxygenated sucrose cutting solution (0–4 °C) containing the following (in mM): sucrose 124, KCl 3, NaHCO_3_ 26, NaH_2_PO_4_ 1.25, glucose 10, sodium pyruvate 3, CaCl_2_ 0.2, MgCl_2_ 3.8 (pH 7.4). Retinal slices 200 μm thick were cut using a manual slicer and were then incubated in artificial cerebrospinal fluid (ACSF) for 40 min prior to recording. ACSF contained the following (in mM): NaCl 125, KCl 3, NaHCO_3_ 26, NaH_2_PO_4_ 1.25, glucose 15, CaCl_2_ 2, MgCl_2_ 1 (pH 7.4).

### Electrophysiological recording and data analysis

The retinal slices were transferred to a chamber, covered with nylon mesh, and continuously perfused with oxygenated (95% O_2_ and 5% CO_2_) ACSF at a rate of 2–3 ml/min. To record mIPSCs, patch pipettes were filled with solution containing the following (in mM): CsCl 150, HEPES 10, EGTA 1, CaCl_2_ 0.1, MgCl_2_ 1, GTP-Na 0.4, and ATP-Mg 4 (pH 7.2 adjusted with CsOH, 275–290 mOsm/l). The neurons were voltage-clamped at −70 mV using an Axopatch-Multiclamp 700B Amplifier (Molecular Devices, Foster City, CA, USA). The sampling frequency was set at 10 kHz, and the filter frequency was 1 kHz. The signals were digitized using a Digidata 1440 A system (Molecular Devices). Data analysis was performed using Clampfit 10.2 (Molecular Devices), Mini Analysis (Synaptosoft), and Origin 8.0 software.

### Drug administration

The tip of a needle was inserted into the superior hemisphere of the eye at a 45° angle through the sclera into the vitreous body. Some rats received an intravitreal injection of 5 μL of PNU-282987 (N-[(3R)-1-azabicyclo[2.2.2]oct-3-yl]-4-chlorobenzamide hydrochloride; 100 μM) or SR95531 (2-[3-carboxypropyl]-3-amino-6-methoxyphenyl-pyridazinium bromide; 100 μM). Other rats were injected with both SR95531 (100 μM) + PNU-282987 (100 μM), and this protocol was repeated weekly thereafter. The control eyes received intravitreal injections of either 5 μL of PBS or 5 μL of SR95531 (100 μM). When recording the mIPSCs, QX314 (lidocaine N-ethyl bromide; 2.0 mM) was added to the pipette solution to block rapid Na^+^ currents. The following drugs were applied using a gravity-fed superfusion system: TTX (1 μM, to abolish spontaneous action potentials), CNQX (10 μM) and AP5 (50 μM) (to inhibit ionotropic glutamate receptors), and strychnine (1 μM, to block glycine receptors). In some slices, MLA (100 nM) was applied 15 min prior to and during PNU-282987 application to block α7-nAChRs and nimodipine (10 μM) was applied 15 min prior to and during PNU-282987 application to block L-type VDCCs. All drugs were purchased from Sigma-Aldrich.

### TUNEL staining

TUNEL staining was performed on 10 μm thick cryosections using an *In Situ* Cell Death Detection Kit. TUNEL signals were visualized using a confocal laser scanning microscope with a 20× objective lens. Fluorescent images of Brn3a-positive cells were obtained simultaneously to confirm the colocalization of RGC markers and TUNEL-positive cells in the GCL. Nuclei were stained with Hoechst 33258.

### Statistical analysis

All data are presented as the mean ± SEM. Student’s *t* test was used to compare the differences in means between two groups, and one-way analysis of variance (ANOVA) with Bonferroni’s post hoc test was used to compare means among multiple groups. The distributions of the amplitudes and inter-event intervals between the events were compared using the Kolmogorov–Smirnov test. In all tests, *p* < 0.05 was considered statistically significant.

## Electronic supplementary material

supplementary information
