# Supplementary material for: Alpha7 nicotinic acetylcholine receptor agonist promotes retinal ganglion cell function via modulating GABAergic presynaptic activity in a chronic glaucomatous model
Source: Sci Rep. 2017 May 11;7:1734. doi: 10.1038/s41598-017-02092-6 (PMC5431927; doi:10.1038/s41598-017-02092-6)
Supplement: Supplementary file 1 — supplementary information [file 41598_2017_2092_MOESM1_ESM.pdf]

Alpha7 nicotinic acetylcholine receptor agonist promotes retinal ganglion cell function via modulating GABAergic presynaptic activity in a chronic glaucomatous model.

Xujiao Zhou[1,2,3], Yun Cheng[1], Rong Zhang[1], Gang Li[1], Boqi Yang[1], Shenghai Zhang[1], Jihong Wu[1,2,3]*

[1] Eye & ENT Hospital, State Key Laboratory of Medical Neurobiology, Institutes of Brain Science and Collaborative Innovation Center for Brain Science, Shanghai Medical College, Fudan University, Shanghai 200032, China

[2] Shanghai Key Laboratory of Visual Impairment and Restoration, Shanghai 200032, China

[3] Key Laboratory of Myopia, Ministry of Health, Shanghai 200032, China

*Correspondence should be addressed to J.W. at Eye & ENT Hospital, State Key Laboratory of Medical Neurobiology, Institutes of Brain Science and Collaborative Innovation Center for Brain Science, Shanghai Medical College, Fudan University; Shanghai Key Laboratory of Visual Impairment and Restoration; Key Laboratory of Myopia, Ministry of Health; #83 Fenyang Road, Shanghai 200032, China. Phone: +86-21-64377134; E-mail: jihongwu@fudan.edu.cn

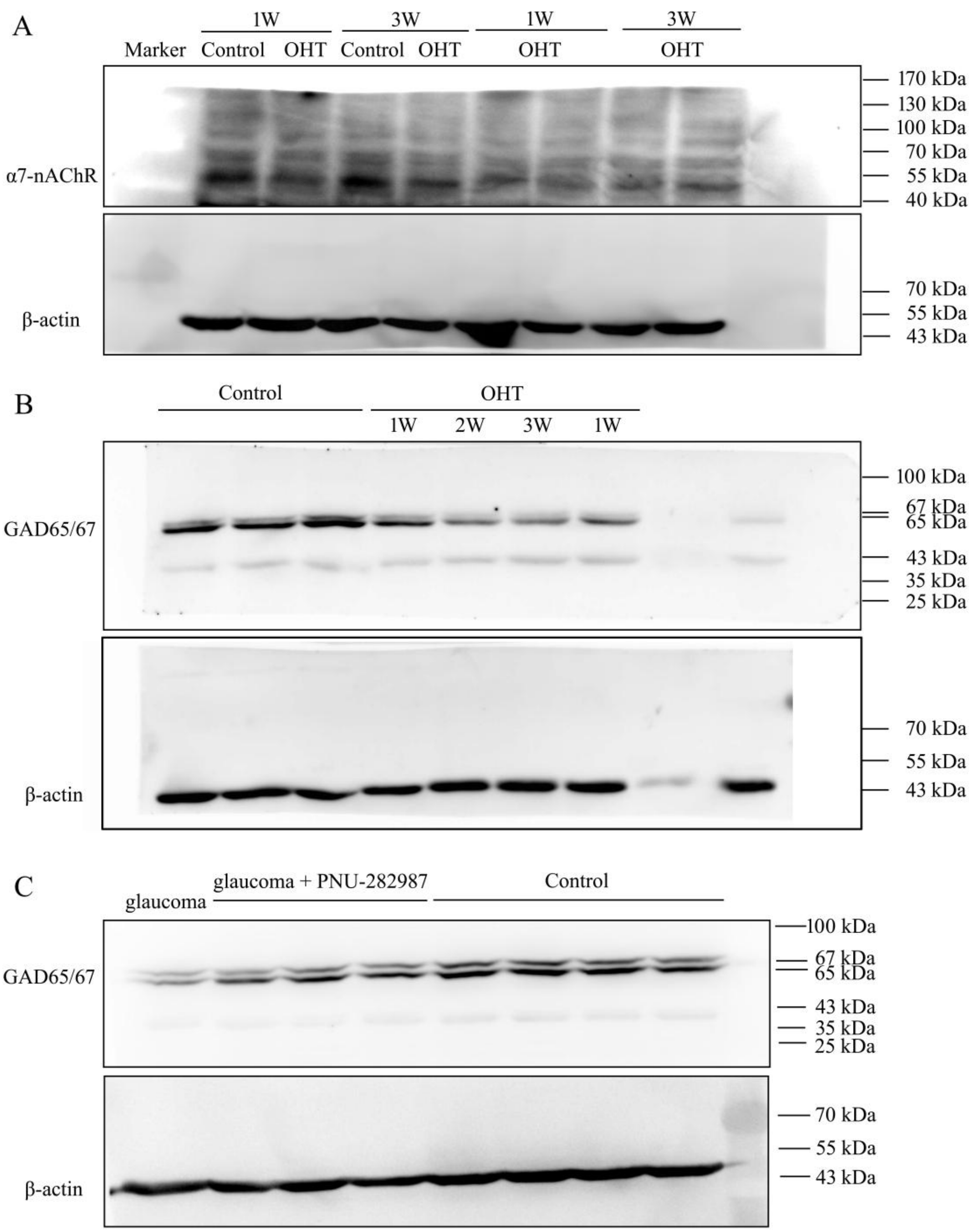

Supplementary Figure Legends

Supplementary Figure S1: The full-length blots of Figure 1C, Figure 4F and Figure 6A. (A) Western blot analysis of proteins expression in control and glaucomatous retinas at 1 week and 3 weeks after EVC. The full-length blots of Figure 1C, showed the expression of α7-nAChR and β-actin. (B) Western blot analysis of proteins expression in control and 1, 2, 3 weeks after EVC. The full-length blots of Figure 4F,

showed the expression of GAD65/67 and β-actin. (C) Western blot analysis of proteins expression in whole retina from control, glaucomatous eyes and PNU-282987 treated eyes. The full-length blots of Figure 6A, showed the expression of GAD65/67 and β-actin. α7-nAChR, α7 nicotinic acetylcholine receptor; w, week; OHT, ocular hypertension; GAD, glutamic acid decarboxylase.
